# Supplementary material for: Limited value of neutrophil-to-lymphocyte ratio and serum creatinine as point-of-care biomarkers of disease severity and infection mortality in patients hospitalized with COVID-19
Source: PLoS One. 2022 Oct 6;17(10):e0275391. doi: 10.1371/journal.pone.0275391 (PMC9536552; doi:10.1371/journal.pone.0275391)
Supplement: Attachment — Submitted filename: Response to Reviewers.docx [file pone.0275391.s002.docx]

Tai-Heng Chen, M.D.

Academic Editor

PLOS ONE

Re: Revision of PONE-D-22-15252 entitled “Neutrophil-to-lymphocyte ratio and serum creatinine as predictors of disease severity and mortality in patients hospitalized with COVID-19”

Dear Dr. Chen,

We thank the editorial team and the reviewers for their very important comments. We have made careful revisions to the original manuscript based on these comments. In the attached letter, we have responded point-by-point to each of the comments that arose during the peer review process. Our responses are in italics against numbered reviewer comments written in plain text.

Sincerely,

Abdisa Tufa

Addis Ababa University, Ethiopia

On behalf of the Authors

Response to Reviewer 1:

1. The predictive value of creatinine levels is unrevealed in this manuscript, it is not consistent with the conclusion.

Response:

*We agree. The predictive value of serum creatinine is incorporated into the manuscript text, figure and table [ Text (line 193-196), Figure 3A and 3B (line 198-199) and table 5 (line 200)]. We have now revised the Conclusion and some aspects of the Result sections.*

2. Based on the ROC curve analysis of NLR, the AUC was only 0.672 for disease severity, with sensitivity of 69.1% and specificity of 60.3%. These results only suggested the ability of NLR to predict disease severity, but not "the most precise biomarker for predicting severity" or "as a best predictor of disease severity" that described in manuscript.

Response:

*We agree and have now carefully revised the Abstract and Main Text to assure that NLR had modest sensitivity and specificity for predicting disease severity and mortality. (indicated in lines 45-46 and 242-243).*

*We also modified the title of the manuscript to address the concern, thus revising it as* *’Neutrophil-to-lymphocyte ratio and serum creatinine as predictors of disease severity and mortality in patients hospitalized with COVID-19*” *(line 1-3).*

3. Other than renal failure, whether other diseases that listed in this manuscript, such as COPD and Cardiovascular diseases could affect the severity of Covid-19 infection?

Response:

*We reported that comorbidities had no clear impact on the severity of COVID-19 or mortality (table 4, line 187-189). Due to the low number of specific diseases among comorbidities, the study did not further investigate the potential effects of COPD, CVD, and other illnesses on study variables. Now, we have added this to the Discussion section as a limitation (line 263-267).*

4. Inclusion/exclusion criteria, diagnostic details should be added in materials and methods part.

Response:

*We agree and have now included the details of the inclusion/exclusion criteria in the manuscript, - ‘2.2. Study subjects’ in the materials and methods part. (Line 90-98).*

Response to reviewer 2:

1. You investigated the role of NLR in COVID-19 which is a simple and practical biomarker that can predict mortality in COVID-19 patients.

***Response****: We thank the Reviewer for the comment.*

2. It would be more appropriate if you can cite the below article

***Response****: Thank you for sharing this important article that we read and cited in the current manuscript accordingly (Line 226-228, reference 32, line 385-387).*

**Journal requirements**

**Response**: Thank you for letting us know the specific PLOS ONE requirements that should be addressed. With this we have now:

- Made necessary revisions to the manuscript to meet PLOS ONE's style.
- Included details regarding participant consent.
- Included Financial Disclosure statement, thus “No authors received funding for this particular study. Centre for Innovative Drug Development and Therapeutic Trials for Africa (CDT-Africa), College of Health Sciences, Addis Ababa University, Addis Ababa supported expenses associated with data and sample collection. Professor Per M. Hellström and Associate professor Dominic-Luc Webb, Gastroenterology and Hepatology Unit, Department of Medical Sciences, Uppsala University, Sweden support all expenses related to laboratory analysis. The funders have no role in the study design, data analysis and decision to publish including preparation of the manuscript”.
- Included Competing Interests, thus “The authors, Abdisa Tufa, Dr. Solomon Genet, Dr. Tewodros Haile Gebremariam and Dr. Yidnekachew Asrat receive their salary from Addis Ababa University. Dr. Tsegahun Manyazewal receives salary from CDT-Africa, Dr. Tsegaye Gebreyes receives salary from Eka Kotobe General Hospital. Tewodros Getinet receives salary from St. Paul’s Hospital Millennium Medical College. Professor Per M. Hellström and Dr. Dominic-Luc Webb receive their salary from Uppsala University”. All authors declare that they have no known competing financial interests or personal relationships that could have appeared to influence the work reported herein.
