# Supplementary material for: Limited value of neutrophil-to-lymphocyte ratio and serum creatinine as point-of-care biomarkers of disease severity and infection mortality in patients hospitalized with COVID-19
Source: PLoS One. 2022 Oct 6;17(10):e0275391. doi: 10.1371/journal.pone.0275391 (PMC9536552; doi:10.1371/journal.pone.0275391)
Supplement: Attachment — Submitted filename: Response to Reviewers.docx [file pone.0275391.s003.docx]

Tai-Heng Chen, M.D.

Academic Editor

PLOS ONE

Re: Revision of PONE-D-22-15252R1 entitled “Limited value of neutrophil-to-lymphocyte ratio and serum creatinine as point-of-care biomarkers of disease severity and infection mortality in patients hospitalized with COVID-19”

Dear Dr. Chen,

We appreciate the editorial staffs and the reviewers' insightful comments. Based on these remarks, we carefully revised the original manuscript. We have addressed each of the criticisms that were made throughout the peer review procedure in detail in the accompanying letter. In contrast to the plain text, numbered reviewer comments, our responses are printed in red ink.

Sincerely,

Abdisa Tufa

Addis Ababa University, Ethiopia

On behalf of the Authors

Response to Reviewer 1:

1. Reviewer #1: Due to the insufficient evidence in the predictive abilities of both N/L ratio and glucose creatinine, their predictive value s in severity and mortality of Covid-19 patients should be seriously considered, as well as the title of revised manuscript.

REPLY: The title has been re-evaluated to give a more humble feature to our findings with the NLR measurements, which seem to be of limited value as studied in our patient cohorts. Likewise, the conclusion has been rectified and the usefullness of the NLR value clearly stated to be of limited value for the prediction of the disease prognosis. For clarity, these requested changes have been underlined.

We have gone through the text body in order to make the whole message of the result section to be in line with conclusion as presented against the background prevailing research data in the discussion.

**Journal requirements**

Please review your reference list to ensure that it is complete and correct.

**Reply**: We have checked the lists of references it is complete and correct.
